# Hamartomas, teratomas and teratocarcinosarcomas of the head and neck: Report of 3 new cases with clinico-pathologic correlation, cytogenetic analysis, and review of the literature

**DOI:** 10.1186/1472-6815-8-8

**Published:** 2008-11-24

**Authors:** Semir Vranic, Samuel K Caughron, Slavisa Djuricic, Nurija Bilalovic, Sadiq Zaman, Ismet Suljevic, William M Lydiatt, Jane Emanuel, Zoran Gatalica

**Affiliations:** 1 Department of Pathology, Clinical Center of the University of Sarajevo, Bosnia and Herzegovina; 2 Department of Pathology, Creighton University Medical Center, Omaha, Nebraska, USA; 3 Mother and Child Health Institute of Serbia "Dr Vukan Cupic", Belgrade, Serbia; 4 Department of Obstetrics and Gynecology, Clinical Center of the University of Sarajevo, Bosnia and Herzegovina; 5 Head and Neck Surgical Oncology, University of Nebraska Medical Center, Omaha, Nebraska, USA; 6 Boystown National Research Hospital, Omaha, Nebraska, USA

## Abstract

**Background:**

Germ-cell tumors (GCT) are a histologically and biologically diverse group of neoplasms which primarily occur in the gonads but also develop at different extragonadal sites in the midline of the body. The head and neck region including the upper respiratory tract is a very rare location for such tumors in both children and adults, which can cause diagnostic and therapeutic difficulties.

**Methods:**

We describe here two new cases of multilineage tumors including sinonasal teratocarcinosarcoma [SNTCS], and congenital oronasopharyngeal teratoma (epignathus) and compare their features with those of a new case of a rare salivary gland anlage tumor [SGAT], an entity for which the pathogenesis is unclear (i.e. hamartoma versus neoplasm). We correlate their presenting clinico-pathological features and compare histologic and cytogenetic features in an attempt to elucidate their pathogenesis and biologic potentials.

**Results and discussion:**

Cytogenetic analysis revealed chromosomal abnormalities only in the case of SNTCS that showed trisomy 12 and 1p deletion. Both cytogenetic abnormalities are characteristically present in malignant germ cell tumors providing for the first time evidence that this rare tumor type indeed might represent a variant of a germ cell neoplasm. The SGAT and epignathus carried no such cytogenetic abnormalities, in keeping with their limited and benign biologic potential.

**Conclusion:**

The comparison of these three cases should serve to emphasize the diversity of multilineage tumors (hamartomas and GCT) of the upper respiratory tract in regards to their biology, age of presentation and clinical outcomes. Malignant tumors of germ cell origins are more likely to affect adults with insidious symptom development, while benign tumors can nevertheless cause dramatic clinical symptoms which, under certain circumstances, can be fatal.

## Background

Upper respiratory tract tumors are relatively common neoplasms whose frequency, distribution, histological type, and clinical behavior are primarily determined by the patient's age, sex and genetic aberrations [1].

Germ-cell tumors are a heterogeneous group of neoplasms that primarily occur in the gonads (both ovaries and testes) but can also occur at extragonadal sites along the midline of the body. The head and neck region including the upper respiratory tract is a rare location for such tumors in both children and adults and only a few cases have been reported in the available literature [1]. Not surprisingly, therefore, the pathologic evaluation and clinical management of these tumors can be very difficult.

Malignant sinonasal tumors are very rare and represent less than 1% of all cancers and approximately 3% of malignancies of the head and neck region [2]. Despite their low frequency, a variety of histological types can be found. Sinonasal teratocarcinosarcoma (SNTCS), also previously described as teratoid carcinosarcoma, malignant teratoma, blastoma, and teratocarcinosarcoma is among the rarest with one study revealing only 1 case (0.5%) of SNTCS among 200 malignant sinonasal tumors [2]. It primarily affects adults (average age 60 years) with only 87 cases reported in the available literature [3-7]. Of the reported cases only five were in patients younger than 20 years which includes one case in a neonate associated with a cleft palate and congenital absence of the ipsilateral Eustachian tube [8,9]. SNTCS is characterized by a histologic combination of malignant teratoma and carcinosarcoma with a triphasic growth pattern including epithelial, mesenchymal, and primitive neuroectodermal components [10]. SNTCS is highly aggressive and occurs mainly located in the nose and paranasal sinuses although tumors occurring in other locations including the nasopharynx and oral cavity have been described [3,8,11] though recently published review of 10 cases with long follow up (up to 372 months) from a single institution revealed significantly better outcome in patients with SNTCS than previously reported [7].

In contrast to malignant germ cell tumors, benign teratomas of the oronasopharyngeal region (so called epignathus) are composed of mature, highly organized structures. They are rare congenital tumors constituting less than 2% of all congenital teratomas, and with an incidence estimated at from 1:35.000 to 1:200.000 live births [12]. The most of epignathi are attached to the base of the skull (hard palate) or to the mandible and are rarely associated with other congenital anomalies [11,13-17].

The outcome and survival of newborns with epignathus are generally unfavorable. The most important factors that determine the outcome include: size of the tumor, degree of facial distortion, airway obstruction, difficulties in management and uni/bidirectional growth pattern of the tumor [18]. Newly developed procedures including ex-utero intra partum (EXIT) procedure may enable survival of newborns with epignathus. The cases are complex, however, with subsequent multidisciplinary surgical management, requiring meticulous planning [19].

Salivary gland anlage tumor [SGAT, also described as congenital pleomorphic adenoma] is a very rare, probably hamartomatous tumor of the nasopharynx of neonates [20]. Due to its histologic features this lesion can be easily misdiagnosed as a neoplasm of a germ cell origin. SGAT was first described by *Har-El et al *[21] in 1985, and a series of nine cases was described by *Dehner et al *in 1994 [20]. Since then 24 cases have been described in the literature including the case we are presenting herein [22]. It typically occurs in neonates in the midline of the nasopharynx with a potential to cause life-threatening airway-obstruction [20].

Cytogenetic studies are particularly useful in determining the germ cell origin of a neoplasm, particularly malignant ones, because they frequently carry a characteristic chromosomal gain of 12 p. In contrast, benign tumors (mature teratomas) show no chromosomal abnormalities [23]. Because of their rarity, cytogenetic and molecular studies of these upper respiratory tract tumors showing multilineage histologic features have generally not been done. Thus their origins remain largely unknown.

Herein, we describe for the first time a case of SNTCS with trisomy 12 with a subclone characterized by an additional deletion of 1 p. We further describe and contrast the case with an epignathus and SGAT that showed no cytogenetic aberrations.

## Methods

Medical records and histopathological reports were retrospectively analyzed. All samples were processed and analyzed using routine pathology techniques, i.e. gross examination, frozen sections (when requested) and formalin-fixation, paraffin-embedding for histologic examination. Hematoxylin and eosin (H&E) stain as well as automated immunohistochemistry (IHC) were used to characterize and diagnose these tumors.

The following primary antibodies were used in IHC: cytokeratin AE1/3, HMB-45, epithelial membrane antigen (EMA), S100, chromogranin, CD99 (DakoCytomation, Carpinteria, CA); cytokeratin 5/6, smooth muscle actin (SMA), alpha-feto protein (AFP), glial fibrillary acidic protein (GFAP), synaptophysin, neuron specific enolase (NSE), myoglobin, myogenin, and p53 (Cell Marque, Hot Spring, AR); vimentin, desmin, bcl-2 (Ventana Medical Systems, Tucson, AZ). Standard indirect biotin-streptavidin detection method with DAB chromagen was used.

### Probe Design and FISH Methods

Fluorescence in situ hybridization (FISH) was performed on 4–5 μm unstained paraffin-embedded tissue sections utilizing the LSI 1p36/1q25 dual color DNA probe (Vysis/Abbott, Downer's Grove, IL) and a dual-color DNA probe mixture combining BAC probes specific for 12p13.2 (RP11-418C2 and RP11-434C1) with CEP 12 (Vysis/Abbott) for ploidy determination. BAC clones were selected according to their genomic location using the UCSC Genome Browser  and labeled by nick translation according to manufacturer's protocol (Vysis/Abbott). Briefly, 200 ng of each rhodamine-5-dUTP labeled BAC clone was precipitated together with 5 times Human Cot-1 DNA (Invitrogen, Carlsbad, CA, USA) and a spectrum green *alpha*-satellite probe for the centromeric region of chromosome 12. Prior to hybridization the slides were pretreated using the VP 2000 automated slide processor (Vysis/Abbott, Inc.) following a modified version of the manufacturer's recommended protocol. Co-denaturation of the DNA probes and patient slide was performed on a HYBrite™ instrument (Vysis/Abbott, Inc.) at 80° C for 5 minutes, followed by an overnight hybridization at 37°C, post-wash in 2 × SSC/0.1% NP-40 at 73° C and 2 × SSC at room temperature for 2 minutes each, and counter-stain with DAPI II. Hybridization signals were assessed in 100 interphase nuclei and images were acquired using the Cytovision Image Analysis System (Applied Imaging, Santa Clara, CA).

### Conventional cytogenetics

The representative tumor samples were disaggregated with scalpels and collagenase and cultured in RPMI-1640 media supplemented with 20% fetal bovine serum and antibiotics for 4–6 days as previously described [24]. Metaphase chromosomes were banded with Wright trypsin and karyotypes were described according to established international guidelines [25].

The case reports were shared with Creighton's University Institutional Review Board; it is however the policy of this Board not to review case reports.

## Results

### Case 1

**Figure 1:**
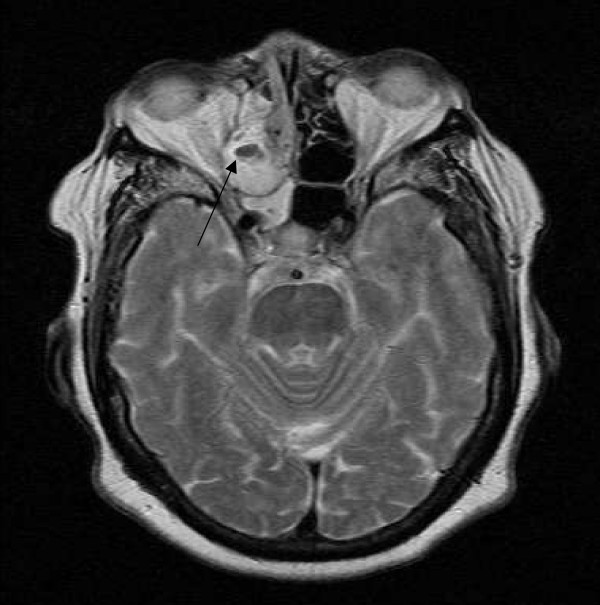
MRI revealed a large polypoid tumor mass eroding through the cribriform plate of the right ethmoid sinus into the cranial cavity.

An 85 year old African American female presented to her primary care physician with complaint of spitting up blood. Imaging studies of the chest did not identify a cause. Five months later she presented with complaints of sinus symptoms and CT scan identified a "large right nasal polyp". Her primary care physician treated her for sinusitis, but three weeks later she presented again complaining of shortness of breath and difficulty moving air through her right nostril. She also reported a bloody nose of three weeks duration and the self discovery of a new nasal polyp one week prior to presentation. On physical exam a large polypoid lesion filled the right naris and MRI identified an extensive right nasal passage mass eroding through the cribriform plate of the right ethmoid sinus into the cranial cavity (Figure 1). A tissue biopsy was obtained and two follow-up biopsies were collected at the request of the pathologist. Arrangements were made for radiation therapy, but one month later, prior to initiation of the therapy, the patient developed progressing neurologic symptoms and subsequently died from a massive intracranial hemorrhage. No autopsy was performed.

#### Pathology findings

**Figure 2:**
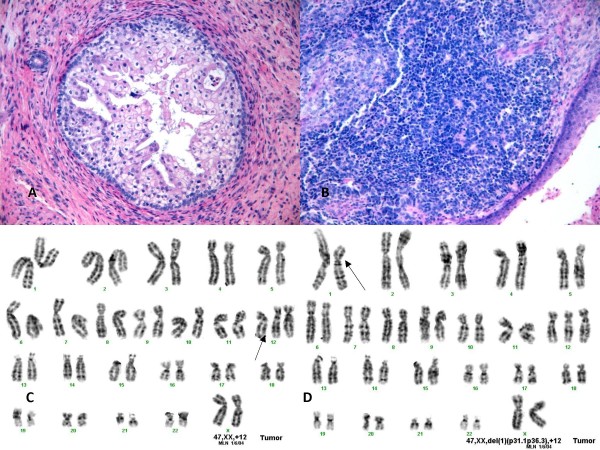
**(A, B): Histologic features of sinonasal teratocarcinosarcoma (Hematoxylin-eosin stain, maginification 20×).** (C): Cytogenetic analysis revealed a hyperploid clone characterized by trisomy 12. (D): An additional subclone contained also a deletion of the short arm of chromosome 1 (del1(p)).

Review of the biopsy materials showed an extensively necrotic tissue with scattered viable epithelial and mesenchymal elements. The different elements blended morphologically into one another. Atypical immature epithelial, mesenchymal and primitive neuroectodermal components were recognized at high magnification (Figure 2). Immunohistochemically, the epithelial components were positive for keratins and EMA, and showed focal positivity for AFP. The mesenchymal and neuroectodermal components were positive for vimentin. HMB45, myoglobin, myogenin and bcl-2 were uniformly negative. Neural markers (S100, GFAP, synaptophysin, chromogranin, NSE) were focally positive in all elements, as were CD99 and desmin. p53 stained approximately 10% of the neuroectodermal components.

Based on the clinical presentation, histologic findings and immunohistochemical characteristics, a diagnosis of teratocarcinosarcoma was made.

#### Cytogenetics

Conventional cytogenetic analysis of 20 metaphase cells identified a hyperdiploid clone characterized by trisomy 12, with an additional subclone characterized by a del(1 p). Karyotype of the tumor was described as: 47, XX, +12[18]/47, idem, del(1)(p31.1p36.3)[2] (see Figure 2, Table 1).

**Table 1:** Results of cytogenetic and FISH analyses of three multilineage tumors

**Case**	**Cytogenetics**	**12 p** **FISH**	**1p36/1q25 FISH**
TERATOCARCINO-SARCOMA	47, XX, +12^18^/ 47, idem, del(1)(p31.1p36.3)	Not performed	Not performed
SGAT	Not performed	Negative	Negative
EPIGNATHUS	Not performed	Unsuccessful	Negative

### Case 2

A 30-year-old G2P1 woman presented at 29 weeks gestation with signs of polyhydramnios. Her first pregnancy had been uneventful. She was referred to the hospital because ultrasound revealed a large mass in the mouth of the live female fetus filling the oral cavity but not protruding out of the mouth. Labor was induced with successful delivery, but postnatal intubation was unsuccessful and the infant died shortly after delivery.

#### Pathology findings

Post mortem examination revealed a female fetus with growth parameters in keeping with 28 weeks of gestation. There was no growth retardation. Petechiae were present on the anterior chest and neck region.

**Figure 3:**
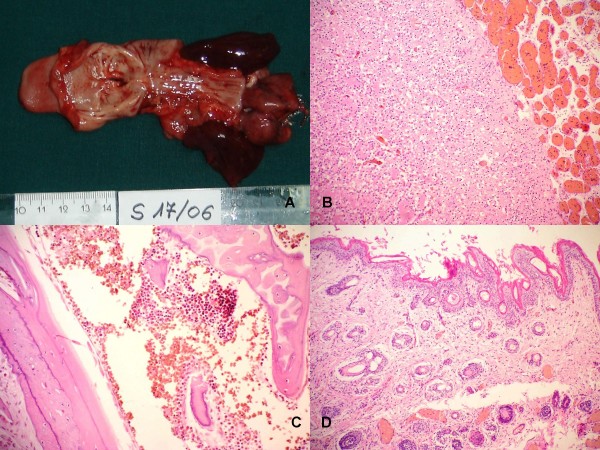
**(A): Gross appearance of the epignathus with tongue-like structures with the blunt border and skin-like surface.** (B-D): Histopathologically, the tumor was composed of different mature teratomatous tissue including brain (B), bone with moderately cellular bone marrow (C), epidermis with numerous hairy follicles and sebaceous glands (D).

A tumor was visible through the slightly opened mouth appearing as a tongue-like structure with a blunt border and skin-like surface. On dissection, the tumor was found to arise from the hard palate and completely filled the oral cavity and upper aero/digestive tract with dilatation of the pharynx (Figure 3A). No transphenoidal intracranial extension of the tumor was found. The tumor measured 5.5 × 4 × 2.5 cm and weighed 36 g. It was completely covered by skin including an area of showing hairy growth. On cut section the tumor was composed of different mature tissue structures including skin, hair, cartilage and centrally located bone

There was no facial dysmorphy, cleft palate or other congenital anomalies. There was no pulmonary hypoplasia.

Histologic examination of the tumor showed variable mature tissue types including bone with bone marrow, hyaline cartilage, epidermis and thick dermis with hairy follicles and sebaceous glands (Figure 3B–D). Subcutaneous fibro-fatty tissue contained skeletal muscle fascicles and salivary glands. Part of the tumor consisted of mature neuroglial tissue covered by leptomeningeal-like layer, rich in blood-filled capillaries. The leptomeningeal-like structures were lined by thin epidermal layer which was in continuity with epidermal skin layer of main part of the tumor. Histological composition of the tumor fully corresponded to a solid mature teratoma (oronasopharyngeal teratoma/epignathus). No atypical, immature or malignant elements were found.

There was extensive intraalveolar, interstitial and subpleural hemorrhage in the immature pulmonary parenchyma of both lungs, along with petechiae, confirming asphyxia as a cause of death.

#### Cytogenetics

Fluorescent in-situ hybridization (FISH) with LSI DNA probes for 1p36 and 1q25 was normal and did not identify chromosomal gains or losses in either. LSI ETV6 (TEL) probe at 12p13 and a homebrew probe set for ETV6 and CEP12 were unsuccessful.

### Case 3

A 12-month old male presented with intermittent airway obstruction and otitis media with bilateral middle ear effusions. The clinical impression was of hypertrophied adenoids, but on closer examination, a mass at the midline posterior nasopharynx was discovered. It was excised and submitted for pathologic examination.

#### Pathology Findings

**Figure 4:**
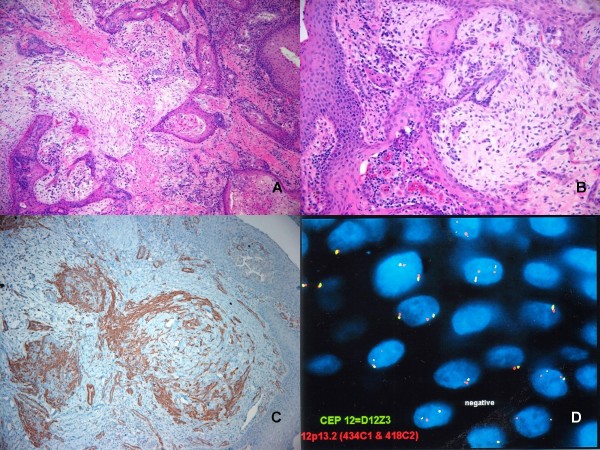
**(A,B): Histological features of salivary gland anlage tumor (Hematoxylin-eosin stain, magnification 10×).** (C): Smooth muscle actin staining highlights both ductal epithelium and myoepithelial cells. (D): FISH analysis revealed 2 copies of 12p13.2 and 12 centromeres with no loss or gain of 1p36 or 1q25.

A 1.7 × 1.0 × 0.6 cm lobulated, light gray to tan, fragment of soft tissue was received for examination. Histopathologic examination revealed a tumor covered by stratified squamous epithelium that extended into the stroma forming a submucosal network of branching tubular structures. These ducts were lined by cuboidal or low columnar epithelium that frequently was transformed into squamous-type lining. The stroma was predominately densely collagenous with myoepithelial cells; focal areas of loose, myxoid stroma (that contained plasma cells) were also present (Figure 4A–B). No cartilaginous or other heterologous elements were found. Immunohistochemical analysis revealed diffuse expressions of cytokeratins (AE1/3 and CK5/6) in the branching ductal epithelium and focal expression among myoepithelial cells. Both structures expressed smooth muscle actin (SMA) and vimentin (Figure 4C).

#### Cytogenetics

Fluorescent in-situ hybridization (FISH) with LSI DNA probes for 1p36 or 1q25 was normal and did not identify chromosomal gain or loss in either region. LSI ETV6 (TEL) probe at 12p13.2 and a homebrew probe set for ETV6 and CEP12 were also normal showing 2 copies per cell nucleus (Figure 4D).

## Discussion

This study reports three new cases of upper respiratory tract tumors, all showing complex, and multilineage histology. The tumors are reported in patients of diverse ages, with significantly varied clinical signs. The presentation of each lesion reflected in part the age of the patient, severity of disease and the biological potential of the tumor. The histologically mature (benign) congenital oronasopharyngeal teratoma (epignathus) caused death from upper airway obstruction in a neonate. The highly malignant SNTCS in an 85-year-female had delayed diagnosis and treatment due to insidious development in the upper respiratory tract and also resulted in the fatal outcome. A hamartomatous SGAT was diagnosed in a 12 month old male and prompt appropriate treatment avoided potentially life-threatening severe respiratory distress and feeding difficulties [26].

The biologic potential of germ cell tumors generally correlates with their gain of chromosomal aberrations. These aberrations are detectable by conventional cytogenetics when fresh tissue is available for cultures. Additionally fluorescent in-situ hybridization (FISH) can be used to detect these abnormalities in formalin fixed paraffin embedded tissues. In our cases we were able to perform cytogenetic analysis on SNCTS and identify trisomy of 12 with a subpopulation of cells showing additional deletion of 1p chromosome. These are the first reported cytogenetic abnormalities found in SNTC.

Trisomy 12, as a primary or secondary event, is a well known cytogenetic abnormality occurring in majority of malignant germ cell tumors [1,27]. The finding of trisomy 12 in our case of SNTCS supports the hypothesis that SNTCS has the germ cell origins though *Salem et al *[6] recently reported three cases of SNTCS with no amplification of chromosome 12p, thus questioning the germ cell origins of SNTCS. We also want to point out that our case of SNTCS bore an additional finding in a form of the deletion of 1p chromosome in a subclone of SNTCS. Loss of 1p is a well characterized genetic feature of embryonal tumors, particularly pediatric germ cell tumors as well as among malignant GCT where it is associated with aggressive clinical course and a poor prognosis [23,28,29]. Notably, loss of 1p (like a gain of chromosome 12p) has also been detected in many other malignant tumor types (e.g. neuroblastoma, ductal breast carcinoma, colorectal carcinoma, malignant melanoma, Wilm's tumor, and endometrial carcinoma), prompting Bussey *et al *[23] to propose that the loss of 1p (or gain of 1q) in case of pediatric germ cell tumors might be indicative of malignancy and also might serve as a prognosticator of a worse outcome. Obviously, additional cases need to be analyzed to confirm our hypothesis since it is also suggested that SNTCS originates from primitive totipotential cells in the olfactory/sinonasal membrane, capable of differentiation into divergent types of somatic cells [3].

In our cases, neither the SGAT nor epignathus carried such cytogenetic aberrations. This result combined with its histopathological and clinical features of SGAT support the thesis that SGAT might be a hamartomatous, developmental disorder rather than true neoplastic lesion. A study of *Bussey et al *[23] concluded that the gonadal and extragonadal teratomas, both mature and immature of children four years and younger, mainly carried normal karyotype without cytogenetic abnormalities though several papers reported diverse cytogenetic abnormalities even in congenital mature teratomas including epignathi [30,31].

Histologically, SNTCS is composed of various tissues including epithelial, mesenchymal, and neural elements including teratoid elements. SNTCS lacks features of embryonal carcinoma, choriocarcinoma, and seminoma [3]. This characteristic distinguishes SNTCS from other malignant germ cell tumors. It is noteworthy that malignant teratomas usually do not exhibit the carcinosarcomatous features, present in SNTCS [3] although it is certainly recognized that a sarcomatous component might be present in some malignant germ cell tumors [32]. Because of its heterogeneous composition, the diagnosis of SNTCS can be quite challenging, particularly if the sampling is not sufficient. Other tumors that may be considered include primarily olfactory neuroblastoma, squamous cell carcinoma, adenocarcinoma, neuroendocrine carcinoma, sarcoma, blastomatous tumors with teratoid features, and craniopharyngeoma [3,33,34]. One feature that seems to be highly suggestive of SNTCS is the presence of the "fetal-appearing" clear cell squamous epithelium [35]. In our opinion this feature also supports the teratoid nature of the tumor.

SGAT is quite rare tumor with only 24 cases (including the case presented in this paper) described since *Dehner et al *[20] introduced this entity. Thus, SGAT is one of the rarest causes of neonatal nasal airway obstruction, and is rarer than congenital tumors like epignathus whose incidence is estimated to be 1:35.000 to 1:200.000 live births [12].

The majority of patients with SGAT present with symptoms (typically respiratory distress and hypoxia) in the neonatal period or first weeks of the life. Our case is the first described with delayed presentation (12 months) and the oldest previously described patient presented with the tumor at the age of three months [22]. For unknown reasons, SGAT is much more prevalent among males and this was supported in our case.

Histopathological features of SGAT include the presence of the stratified squamous epithelium that extends into a loose, myxoid stroma forming a submucosal network of branching tubular structures (tubular to cord-like epithelial structures). Areas with necrosis and cystic degeneration might also be present [22]. Only one case with a widespread necrosis and large cyst formations has been described to date [36]. Many other hamartomatous and teratoid lesions may arise in sinonasal region including mesenchymal, adenomatoid, and glandular hamartomas depending on the predominance of the specific structures [2].

SGAT exhibits benign behavior and no recurrences after complete surgical resection were reported in the literature [26]. It may be accompanied by other midline developmental disorders in the head and neck region including dermoid sinus, nasal glioma, and thyroglossal duct cyst [22].

Teratomas have origins in totipotential germ cells and along with neuroblastoma they are the most common congenital tumors. Nevertheless, head and neck (oronasopharyngeal) teratomas are exceptionally rare comprising less than 2% of all congenital teratomas [37]. They are characteristically composed of different tissue types from different germ cell layers. Teratomas are histologically classified as either mature or immature, where immature elements consist mainly of primitive neuroglial tissue and neuroepithelial rosettes. Histopathological analysis of our tumor revealed no immature neural or other tissue elements, thus classifying it as a mature (benign) teratoma (epignathus). This is in concordance with other studies since the most epignathi are benign with extremely rare malignant alteration [38].

Our case of epignathus provides lessons for both diagnosis and care of patients with this tumor. Uncharacteristically, the tumor was not associated protrusion from the oral cavity, intracranial extension or other midline anomalies including cleft palate as the most common anomaly associated with epignathus [13,17]. Nevertheless the growth pattern was very specific with the tumor filling the oral cavity and upper aerodigestive tract. In our case this one feature prevented successful intubation after birth and caused death from what might otherwise have been considered a benign neoplasm. If the diagnosis can be established, future cases may consider alternative treatment approaches including a recently developed EXIT procedure to allow for survival of the newborn. Multidisciplinary approach should always be used, although successful treatment and outcome from these lesions is likely to remain challenging [39].

## Conclusion

The comparison of these three cases should serve to emphasize the diversity of multilineage tumors (hamartomas and GCT) of the upper respiratory tract in regards to their biology, age of presentation and clinical outcomes. Malignant tumors of germ cell origins are more likely to affect adults with insidious symptom development, while benign tumor can nevertheless cause dramatic clinical symptoms which, under certain circumstances, can be fatal.

## Competing interests

The authors declare that they have no competing interests.

## Authors' contributions

SV carried out autopsy, participated in diagnostics, conceived the study design, wrote and drafted the manuscript. SKC, SZ, IS, WML, and JM participated in diagnostics, wrote and approved the final manuscript. SD and NB carried out autopsy, wrote and approved the final manuscript. ZG participated in diagnostics, conceived the study design, wrote and approved the final manuscript. All authors read and approved the final manuscript.

## Pre-publication history

The pre-publication history for this paper can be accessed here:


